# Vitamin E in diabetes-associated cognitive dysfunction: mechanistic rationale, current evidence and translational gaps

**DOI:** 10.3389/fnut.2026.1901124

**Published:** 2026-10-28

**Authors:** Wenting Geng, Yihan Fan, Peng Li, Renjun Lv, Di Zhang, Hua Shao

**Affiliations:** 1 College of Traditional Chinese Medicine, Shandong University of Traditional Chinese Medicine, Jinan, Shandong, China; 2 Department of Neurology, The First Affiliated Hospital of Shandong First Medical University and Shandong Provincial Qianfoshan Hospital, Shandong Institute of Neuroimmunology, Jinan, Shandong, China; 3 Medical Department, Shandong Academy of Occupational Health and Occupational Medicine, Shandong First Medical University & Shandong Academy of Medical Sciences, Jinan, Shandong, China; 4 Department of Neurology, Shandong Provincial Hospital Affiliated to Shandong First Medical University, Jinan, Shandong, China

**Keywords:** diabetes-associated cognitive dysfunction, insulin resistance, neuroinflammation, neurovascular dysfunction, oxidative stress, synaptic plasticity, vitamin E

## Abstract

Diabetes-associated cognitive dysfunction (DACD) is an increasingly recognized complication of diabetes mellitus and involves multiple interacting processes, including oxidative stress, neuroinflammation, synaptic dysfunction, impaired insulin signaling, and neurovascular dysfunction. Vitamin E, a lipid-soluble antioxidant comprising tocopherols and tocotrienols, has attracted interest because several of its biological actions are mechanistically relevant to these pathways. This mini-review provides a focused overview of the mechanistic rationale, current experimental and clinical evidence, and major translational gaps concerning vitamin E in DACD. Preclinical studies suggest that specific vitamin E isoforms and preparations may modulate oxidative stress and lipid peroxidation, inflammatory signaling and microglial activation, synaptic plasticity-related pathways, and insulin responsiveness, and may also influence neurovascular processes. However, much of this evidence derives from animal models, cellular systems, peripheral tissues, or disease settings other than DACD and therefore provides mechanistic rather than clinical efficacy evidence. Direct clinical evidence in patients with formally diagnosed DACD remains lacking. Small intervention studies in patients with type 2 diabetes have reported inconsistent cognitive outcomes, whereas observational associations between dietary vitamin E intake and cognition are largely derived from non-DACD populations and cannot establish causality. High-dose supplementation may also entail safety concerns. Thus, current evidence supports the biological plausibility of vitamin E as a candidate for further investigation but does not establish its preventive or therapeutic efficacy in DACD. DACD-specific randomized controlled trials are needed to determine whether particular vitamin E isoforms, doses, or intervention durations provide clinically meaningful cognitive benefits and to establish long-term safety.

## 1 Introduction

Diabetes mellitus is a major public health concern worldwide. Epidemiological data from mainland China indicate that diabetes prevalence has continued to rise in recent years, with more than 90% of adult cases being type 2 diabetes mellitus (T2DM) (1). T2DM is closely associated with cognitive impairment, with patients having an approximately 1.5- to 2.0-fold higher risk than individuals without diabetes (2). Diabetes-associated cognitive dysfunction (DACD) has become a chronic condition that substantially impairs quality of life and poses a major health burden in older adults. The pathogenesis of DACD is a complex process involving multiple factors and pathways. Key mechanisms include oxidative stress, neuroinflammation, impaired synaptic plasticity, insulin resistance, and neurovascular dysfunction (3–5). According to disease severity, it can be classified as mild cognitive impairment and dementia. “Cognitive impairment” refers to measurable cognitive deficits, whereas “cognitive decline” denotes progressive worsening of cognitive performance over time. “Dementia” represents the more severe clinical stage of DACD. Patients with DACD commonly exhibit impaired learning and memory, diminished executive function, and attentional deficits. Epidemiological data indicate that dementia has emerged as a leading cause of death among diabetic patients in some countries or regions, underscoring an urgent public health concern (6). Currently, DACD management relies primarily on glycemic control, while specific preventive and therapeutic strategies remain limited.

Dietary factors may influence cognitive health and the risk of cognitive impairment. Numerous studies have shown that certain nutrients can modulate the risk of cognitive impairment and dementia. Vitamin E may exert neuroprotective effects through antioxidant and anti-inflammatory actions, membrane stabilization, regulation of synaptic and insulin-related signaling, and modulation of neurovascular function. Accumulating preclinical evidence, together with limited clinical evidence, suggests that vitamin E may exert neuroprotective effects and ameliorate cognitive impairment (7, 8). This review considers DACD broadly across diabetes mellitus, with particular emphasis on T2DM. Relevant mechanistic evidence from both type 1 diabetes mellitus (T1DM)-like and T2DM models is included. In this mini-review, we summarize evidence on the relevance of vitamin E to DACD, focusing on mechanisms, clinical evidence, safety, and translational gaps. A focused, non-systematic literature search of PubMed and Web of Science was conducted through June 2026. Search terms combined “vitamin E,” “tocopherol,” or “tocotrienol” with terms related to diabetes and cognitive outcomes, supplemented by pathway-specific terms such as oxidative stress, neuroinflammation, synaptic plasticity, insulin signaling, neurovascular dysfunction, and blood–brain barrier. Diabetic models with cognitive assessment were prioritized, while indirect experimental models were used to support mechanistic plausibility. Human intervention and observational studies were evaluated separately, with evidence weighted by study design, directness to DACD, and cognitive outcomes. Recent reviews have summarized the relationship between vitamin E and cognitive health, including the roles of different tocopherol and tocotrienol forms (9, 10). This review focuses specifically on DACD, evaluates the strength and clinical relevance of current evidence, and highlights key knowledge gaps and priorities for future translational research.

## 2 Vitamin E dietary sources, forms, bioavailability, and nutritional relevance in diabetes

Vitamin E is a fat-soluble molecule found in plants and many foods characteristic of the Mediterranean diet. Vegetable oils, especially wheat germ oil (20.3 mg α-tocopherol/tablespoon) and sunflower oil (5.6 mg α-tocopherol/tablespoon), are among the richest dietary sources of vitamin E. Additional significant sources include nuts, seeds, green leafy vegetables, tomatoes, and mangoes. According to the Office of Dietary Supplements of the National Institutes of Health, the recommended dietary intake of vitamin E for adults is approximately 15 mg/day as α-tocopherol (11). Vitamin E exists in eight naturally occurring forms, including four tocopherols (α-, β-, γ-, and δ-tocopherol) and four tocotrienols (α-, β-, γ-, and δ-tocotrienol) (12). These isoforms differ in their absorption, distribution, tissue retention, metabolism, and elimination. Hepatic α-tocopherol transfer protein (α-TTP) preferentially binds α-tocopherol and facilitates its incorporation into circulating lipoproteins, thereby promoting higher plasma concentrations and greater tissue retention (13). These pharmacokinetic properties underlie the higher conventional vitamin E activity assigned to α-tocopherol and contribute to its predominant use in dietary supplements (14). In contrast, γ-tocopherol and other isoforms have lower affinity for α-TTP and undergo more extensive hepatic metabolism through ω-hydroxylation followed by β-oxidation, with subsequent urinary or biliary excretion. Consequently, γ-tocopherol generally exhibits lower circulating concentrations and tissue retention than α-tocopherol, which may partly explain its lower conventional vitamin E activity (15). α-Tocopherol appears to be the predominant tocopherol in the human brain; in postmortem tissue samples from four cortical and subcortical regions, it accounted for approximately 62–72% of the measured tocopherol concentrations (16). Human studies have also detected both α- and γ-tocopherol in cerebrospinal fluid (CSF), with CSF concentrations correlating with their corresponding serum concentrations (17). Experimental studies further indicate that orally administered tocotrienols can reach brain tissue; however, their cerebral concentrations appear to be relatively low compared with α-tocopherol in animal models (18). Thus, blood–brain barrier permeability and cerebral exposure among different isoforms remain incompletely characterized, particularly for tocotrienols. Studies have reported that serum vitamin E concentrations in patients with T2DM were lower than those in individuals without diabetes (19). Several factors may contribute to this reduction. Chronic hyperglycemia is associated with increased oxidative stress and lipid peroxidation, which may increase vitamin E utilization as a lipid-soluble antioxidant (20). In addition, because vitamin E is transported mainly in circulating lipoproteins, diabetes-related alterations in lipid and lipoprotein metabolism may affect its transport, distribution, and measured serum concentration (21). Therefore, serum vitamin E concentrations should be interpreted in the context of circulating lipid levels. Hypercholesterolemia or hypertriglyceridemia may increase the lipoprotein pool available for α-tocopherol transport and consequently elevate its absolute serum concentration without necessarily indicating greater tissue vitamin E availability or nutritional status (22). Lipid-adjusted indices, such as the α-tocopherol/total cholesterol ratio or the α-tocopherol/(cholesterol + triglycerides) ratio, may provide complementary information to absolute α-tocopherol concentrations (23). Lower serum vitamin E concentrations have been associated with greater insulin resistance and increased oxidative stress (24). Vitamin E intake may modestly improve glycemic control and insulin resistance in diabetic patients, particularly in lowering glycated hemoglobin levels (25).

## 3 Mechanistic rationale for vitamin E in DACD

The pathogenesis of DACD involves interconnected metabolic, neurovascular, inflammatory, and neuronal disturbances. Chronic hyperglycemia and insulin resistance promote oxidative stress, lipid peroxidation, mitochondrial dysfunction, and advanced glycation end-product accumulation (26). Oxidative stress may further contribute to inflammatory signaling and microglial activation (27), whereas neuroinflammation and impaired insulin signaling are linked to neuronal injury, reduced neurotrophic support, abnormal amyloid-β (Aβ) and tau homeostasis, synaptic dysfunction, and cognitive impairment (28–30). In parallel, neurovascular dysfunction may impair cerebral perfusion and thereby further contribute to cognitive impairment (4). These mechanisms form an interconnected pathological network rather than isolated pathways. As discussed below, vitamin E may influence multiple nodes within this network through its potential antioxidant, anti-inflammatory, insulin-sensitizing, and synaptoprotective effects as well as possible modulation of neurovascular function. Evidence directly derived from diabetic or DACD-specific models is prioritized in this review. Findings from aging and Alzheimer’s disease models, peripheral tissue studies, and *in vitro* cell culture systems are considered indirect mechanistic evidence because they share some pathological features with DACD. Table 1 summarizes the preclinical evidence discussed below, including experimental models, vitamin E formulations, doses, intervention durations, main outcomes, and their direct or indirect relevance to DACD.

**Table 1:** Preclinical evidence relevant to vitamin E and diabetes-associated cognitive dysfunction.

Experimental models	Vitamin E form	Dose	Duration	Main outcomes	Relevance to DACD	Reference
aged mouse models	Vitamin E (form not specified)	100, 200, and 400 mg/kg/day	4 weeks	↑ hippocampal SIRT1, Nrf2, and Calstabin2 expression; Improved Y-maze working memory and spatial cognition	Indirect (aging models)	(35)
STZ-induced diabetic male Wistar rats with learning and memory impairment (T1DM-like)	Vitamin E (form not specified)	100 mg/kg/day	7 weeks	Improved spatial learning and memory; ↓ lipid peroxidation; restored glutathione levels in the hippocampus and frontal cortex	Indirect (T1DM-like diabetic models with cognitive assessment)	(36)
Aged rat models	RRR-α-tocopherol acetate	2.0/5.0 g vitamin E/kg diet	3 months	↑ hippocampal mitochondrial respiration and complex I/IV activity; ↓ mitochondrial ROS and oxidative damage	Indirect (aging models)	(37)
STZ-induced diabetic male Wistar rats with learning and memory impairment (T1DM-like)	Tocotrienol	25, 50, or 100 mg/kg/day	10 weeks	Improved spatial learning and memory; ↓ TNF-α, IL-1β, NF-κB activation, and caspase-3 activity	Indirect (T1DM-like diabetic models with cognitive assessment)	(40)
Alloxan-induced diabetic model (cutaneous wound tissue)	γ-tocopherol	35 mg/kg, 5 times/week	2 weeks	↓ NF-κB、IL-1β and TNF-α	Indirect (peripheral-tissue evidence)	(41)
HFD/STZ diabetic model (skeletal muscle)	Tocotrienol-rich fraction (TRF)	100/300 mg/kg, 5 times/week	12 weeks	↓ NF-κB, IL-6, TNF-α; Improved insulin resistance; enhanced IRS-1/Akt/GLUT4 and AMPK/SIRT1/PGC1α signaling	Indirect (peripheral-tissue evidence)	(42)
Primary microglial cells	α-tocopherol	10/20/100 μg/mL	48 h for morphology; up to 72 h for adhesion markers	Promoted ramification/resting-like morphology of microglia; ↓ ICAM-1, LFA-1, and VLA-4 expression	Indirect (cellular evidence)	(47)
LPS-activated microglial cellular model	DL-α-tocopherol acetate	100 μM	1 h pretreatment before LPS; 24 h total incubation	Preserved ramified/resting-like microglial morphology; ↓ LPS-induced migration and TNF-α; ↑ IL-10; ↓ p-AKT.	Indirect (cellular evidence)	(48)
icv-STZ-induced sporadic Alzheimer’s disease-like model	α-tocopherol	150 mg/kg/day	7 days pre-treatment + 14 days post-treatment	Restored hippocampal LTP; decreased Aβ plaque accumulation and neuronal loss.	Indirect (other neurodegenerative models)	(52)
Hyperoxia-induced oxidative stress model	α-tocopherol	250 mg/100 g diet	19 weeks	Prevented oxidative stress-induced loss of SNARE proteins involved in synaptic function and neurotransmission.	Indirect (other neurodegenerative models)	(53)
Vitamin E-deficient C57BL/6 mice	α-tocopherol deficiency	Vitamin E-deficient diet (AIN-76A)	5 months	Induced hippocampal axonal degeneration; decreased CRMP-2 expression	Indirect	(54)
Male adult rats fed a high-fat diet	α-tocopherol	500 IU/kg diet	2 months	Restored hippocampal BDNF expression and synaptic plasticity-related signaling.	Indirect (other neurodegenerative models)	(55)
Male C57BLKS/J-Lepr^db/db^ mice	Tocotrienol-rich fraction (TRF)	50 mg/kg/day	2 weeks	Improved insulin sensitivity and glucose metabolism through activation of PPAR signaling and metabolic gene regulation.	Indirect (diabetic models without cognitive assessment)	(57)
Insulin-resistant human SK-N-SH neuronal cells induced by chronic hyperinsulin exposure	Tocotrienol-rich fraction (TRF)	200 ng/mL	24 h	↑ AKT phosphorylation and glucose uptake; ↓ ROS production and GSK3β and TAU expression	Indirect (cellular evidence)	(58)
STZ-induced diabetic male Wistar rats with cognitive impairment (T1DM-like)	α-Tocopherol acetate	100 mg/kg/day	7 weeks	Improved memory performance; partially reversed diabetes-induced alterations in NO-producing neurons	Indirect (T1DM-like diabetic models with cognitive assessment)	(61)
Aged male Wistar rats (23 months old)	Tocotrienol mixture (α-, β-, γ-, and δ-tocotrienols)	2.5 g/kg diet	3 weeks	Improved learning and memory; α- and γ-tocotrienols were detected in brain tissue; did not restore age-related reductions in BBB-associated proteins (claudin-5, occludin, and JAM)	Indirect (aging models)	(62)

Evidence from aging, cellular, peripheral-tissue, other neurodegenerative, or diabetic models without cognitive assessment is considered indirect mechanistic evidence. Diabetic models with cognitive impairment were not considered equivalent in translational relevance. Chemically induced T1DM-like models were considered DACD-relevant preclinical evidence but were interpreted cautiously because they do not fully reproduce the metabolic, vascular, and age-related features of T2DM-associated cognitive dysfunction.

### 3.1 Vitamin E and oxidative stress

Although the brain accounts for only 2% of body weight, it consumes approximately 20% of total oxygen and contains abundant polyunsaturated fatty acids that are highly susceptible to oxidation. In addition, the brain has relatively low glutathione levels and reduced activities of antioxidant enzymes, including catalase and glutathione peroxidase, compared with other organs. Consequently, its antioxidant defense capacity is relatively limited, making it particularly vulnerable to oxidative stress (31). Numerous studies have demonstrated that oxidative stress contributes to the initiation and progression of neurodegenerative diseases. Chronic hyperglycemia may promote oxidative stress. In patients with diabetes, levels of advanced glycation end-products (AGEs) are elevated. Binding of AGEs to their receptors activates inflammatory signaling and increases reactive oxygen species (ROS) generation. Excessive ROS accumulation leads to lipid peroxidation, mitochondrial dysfunction, and disordered energy metabolism, thereby accelerating cerebral microvascular injury and neuronal apoptosis (32). Vitamin E counteracts Aβ-induced neurotoxicity through free radical scavenging (33). Vitamin E effectively prevents lipid peroxidation of the plasma membrane, protects neuronal membranes, and preserves the structural integrity of mitochondrial membranes (34). Animal studies further support the antioxidant properties of vitamin E. Sirtuin 1 (SIRT1), nuclear factor erythroid 2-related factor 2 (Nrf2), and Calstabin2 constitute a coordinated intracellular antioxidant defense network. They detect oxidative stress, activate antioxidant responses, and maintain cellular integrity. In aged mouse models, vitamin E supplementation significantly upregulated the cerebral expression of SIRT1, Nrf2, and Calstabin2 in a dose-dependent manner, with the greatest effect observed at 400 mg/kg (35). However, doses used in animal studies cannot be directly extrapolated to humans because of interspecies differences in metabolism, absorption, bioavailability, tissue distribution, and pharmacokinetics. Therefore, these preclinical doses should be interpreted as mechanistic rather than clinically applicable dose estimates. Closer to the diabetic context, in streptozotocin (STZ)-induced diabetic rats, vitamin E treatment improved Morris water maze performance, reduced lipid peroxidation, and restored glutathione levels in the hippocampus and frontal cortex (36). Notably, chemically induced insulin-deficient models do not fully reproduce the complex metabolic features of T2DM-associated DACD, limiting their translational relevance to cognitive dysfunction in older adults with T2DM. Moreover, vitamin E supplementation can increase vitamin E levels in mitochondria, preserve the activities of respiratory chain complexes I and IV, and maintain efficient mitochondrial respiration. These effects may reduce mitochondrial superoxide and hydrogen peroxide production, thereby limiting oxidative damage to mitochondrial DNA (37, 38). However, these findings provide indirect mechanistic support and require further validation in DACD models.

### 3.2 Vitamin E anti-inflammatory effects

Chronic hyperglycemia is a potent activator of the nuclear factor kappa B (NF-κB) pathway. In db/db mice, the diabetic hyperglycemic environment stimulates microglial ROS production and activates the NF-κB/NOD-like receptor protein 3 (NLRP3) signaling pathway, resulting in increased production of inflammasome-related mediators such as interleukin-1β (IL-1β) and interleukin-18 (IL-18) (39). Inhibition of NF-κB signaling represents a major mechanism underlying the anti-inflammatory effects of tocopherols and tocotrienols. In STZ-induced diabetic rats with cognitive impairment, tocotrienol improved spatial learning and memory while suppressing tumor necrosis factor-α (TNF-α), IL-1β, NF-κB activation, and caspase-3 activity in the brain (40). Indirect mechanistic evidence relevant to DACD has also been obtained from peripheral tissues. In alloxan-induced diabetic mice, γ-tocopherol supplementation reduced the protein levels of NF-κB, IL-1β, and TNF-α in cutaneous wound tissue (41), whereas tocotrienol-rich fraction (TRF) reduced NF-κB, interleukin-6 (IL-6), and TNF-α levels in skeletal muscle of high-fat diet (HFD)/STZ-induced T2DM mice (42). Different vitamin E forms inhibit NF-κB activation through distinct mechanisms. δ-Tocotrienol induces the expression of the NF-κB negative regulator A20 by modulating sphingolipid metabolism, thereby suppressing transforming growth factor-β (TGF-β)-activated kinase 1 (TAK1) phosphorylation and NF-κB activation (43). γ-Tocotrienol inhibits TNF-induced NF-κB activation by inhibiting IκB kinase (IKK) activation (44).

Microglia adopt dynamic and context-dependent states in response to metabolic, inflammatory, and tissue-specific signals, involving coordinated changes in transcriptional profiles, cellular functions, and morphology (45, 46). In the diabetic brain, persistent metabolic and inflammatory stress may promote microglial states characterized by enhanced production of pro-inflammatory mediators. In primary microglial cultures, α-tocopherol induced a more ramified morphology and reduced the expression of adhesion molecules, including lymphocyte function-associated antigen 1 (LFA-1), very late antigen 4 (VLA-4), and intercellular adhesion molecule 1 (ICAM-1) (47). More recent evidence further showed that α-tocopherol attenuated lipopolysaccharide (LPS)-induced microglial responses by reducing cell migration and TNF-α production, increasing interleukin-10 (IL-10) production, and modulating phosphoinositide 3-kinase (PI3K)/protein kinase B (Akt) signaling (48). Additionally, α-tocopherol suppresses microglial activation through inhibition of p38 mitogen-activated protein kinase and NF-κB signaling, potentially protecting neurons from inflammatory injury (49). Together, these findings suggest that α-tocopherol may modulate microglial inflammatory responses, although its direct effects on microglia in DACD remain to be established.

### 3.3 Vitamin E and synaptic plasticity

Synaptic plasticity is the cellular basis of learning and memory. The interaction among neuroinflammation, neurotrophic factors, and synaptic proteins may contribute to impaired synaptic plasticity, which is considered a neuropathological mechanism underlying DACD. Patients with diabetes experience chronic inflammation, which may activate microglia and astrocytes. Inflammatory mediators released by these cells disrupt the neuronal microenvironment, promote synaptic protein degradation, and impair synaptic function. In diabetic mice, reduced hippocampal dendritic spine density, synapse number, and presynaptic mitochondrial density have been observed, accompanied by impaired learning and memory (50). Compared with controls, diabetic mice also exhibited reduced hippocampal levels of brain-derived neurotrophic factor (BDNF), synaptophysin (SYN), and postsynaptic density protein 95 (PSD-95) (51). Preclinical studies, mainly involving α-tocopherol, suggest potential synaptoprotective effects. In a sporadic Alzheimer’s disease-like model induced by intracerebroventricular STZ, α-tocopherol improved synaptic plasticity and cognitive performance by restoring long-term potentiation (LTP), reducing Aβ deposition, and decreasing neuronal death (52). α-Tocopherol also preserves soluble N-ethylmaleimide-sensitive factor attachment protein receptor (SNARE) components, including synaptobrevin and synaptotagmin on synaptic vesicles and syntaxin-1 on the presynaptic membrane, thereby supporting neurotransmission (53).

Collapsin response mediator protein-2 (CRMP-2) is essential for maintaining axonal structure and transport. Axonal degeneration is associated with reduced CRMP-2 expression and abnormal CRMP-2 modification. Total CRMP-2 expression was reduced in the cerebral cortex and hippocampus of vitamin E-deficient mice. A similar decline is also observed in the cortex of normal aged mice. This study suggests that α-tocopherol deficiency may independently contribute to axonal degeneration in the hippocampal CA1 region, apart from the aging process, representing a potential early mechanism of cognitive dysfunction (54). These findings suggest a potential role of α-tocopherol status in maintaining axonal integrity. Beyond structural synaptic protection, α-tocopherol may enhance BDNF expression, thereby supporting synapse formation and cognitive processes. In a model induced by a diet high in saturated fat, α-tocopherol reversed oxidative stress-induced reductions in BDNF, synapsin I, and cyclic adenosine monophosphate (cAMP) response element-binding protein (CREB) to normal levels, while also preserving the activation of synapsin I and CREB (55). Overall, these α-tocopherol studies suggest potential effects on synaptic integrity and cognitive function through its antioxidant effects, regulation of neurotransmitter release, maintenance of membrane fluidity, and enhancement of LTP. However, direct evidence that vitamin E supplementation restores synaptic plasticity in diabetic models remains limited.

### 3.4 Vitamin E and insulin resistance

Insulin resistance is a central pathophysiological feature of T2DM and a key mechanism implicated in DACD (56). Insulin resistance disrupts cerebral insulin signaling. Because insulin signaling pathways regulate Aβ production and tau phosphorylation, impairment of this pathway may contribute to cognitive dysfunction. Specific tocotrienol preparations may modulate insulin-responsive pathways, although their downstream effects on Aβ and tau pathology remain unclear. In db/db mice, TRF lowered fasting blood glucose levels and improved performance in oral glucose tolerance tests and insulin tolerance tests. Tocotrienols improve peripheral insulin sensitivity and glucose metabolism by activating peroxisome proliferator-activated receptors (PPARs). Specifically, α-tocotrienol and γ-tocotrienol selectively activate PPARα, whereas δ-tocotrienol activates PPARα, PPARγ, and PPARδ (57). At the neuronal level, TRF alone increased Akt phosphorylation and reduced ROS, GSK3β, and TAU mRNA expression in insulin-resistant SK-N-SH cells, but did not restore IR, PI3K, GLUT3, or GLUT4 expression (58). Combined vitamin D and TRF treatment further increased GLUT4 expression and Akt phosphorylation and reduced ROS, GSK3β, and TAU expression. Because tau phosphorylation was not directly assessed, these changes reflect transcriptional responses rather than altered tau phosphorylation. Overall, TRF appears to modulate selected downstream insulin-responsive and redox pathways without fully restoring neuronal insulin signaling. In HFD/STZ-induced T2DM mice, TRF improved skeletal-muscle insulin resistance and mitochondrial metabolism, with modulation of GLUT4 translocation and AMP-activated protein kinase (AMPK)/SIRT1/peroxisome proliferator-activated receptor γ coactivator 1α (PGC-1α) signaling, together with reduced oxidative and inflammatory markers (42). These findings provide peripheral metabolic and cellular mechanistic evidence, but their relevance to cerebral insulin signaling and DACD requires further validation.

### 3.5 Vitamin E and neurovascular dysfunction

Neurovascular dysfunction is an important component of DACD and interacts with metabolic, oxidative, and inflammatory disturbances. Chronic hyperglycemia and insulin resistance can impair endothelial function and cerebral microvascular integrity, thereby reducing vascular reactivity and compromising blood–brain barrier (BBB) function. In T2DM rats, reduced hippocampal cerebral blood flow and lower vascular endothelial growth factor expression were associated with poorer spatial learning and memory, supporting a role of cerebral hypoperfusion in DACD (4). In Goto–Kakizaki rats, reduced nitric oxide (NO) bioavailability was accompanied by weaker hippocampal microvascular responses and impaired spatial memory, indicating that disrupted neurovascular coupling may contribute to early cognitive dysfunction in T2DM (59). Hyperglycemia may also damage cerebral microvascular endothelial cells through PKC-dependent oxidative signaling (60).

In STZ-induced diabetic rats, α-tocopherol acetate improved memory performance and partly reversed diabetes-related alterations in NO-producing neurons (61), suggesting a possible interaction between vitamin E and NO-related neuronal signaling. However, cerebral perfusion and neurovascular coupling were not directly examined. In aged rats, a tocotrienol mixture improved learning and memory, while α- and γ-tocotrienols were detected in brain tissue after supplementation (62). Changes in BBB-associated proteins were also observed in this model, although tocotrienol supplementation did not fully restore their age-related reduction. Together, these findings suggest that neurovascular pathways may represent an additional mechanistic link between vitamin E and cognitive function; however, direct evidence that vitamin E improves cerebral perfusion, BBB integrity, or neurovascular coupling in DACD remains scarce.

## 4 Limited clinical evidence and safety considerations

Vitamin E has different clinical implications according to nutritional status and the level of exposure. Correction of confirmed deficiency aims to restore adequate vitamin E status, although its effect on DACD has not been established. Habitual dietary intake represents long-term nutritional exposure and is supported mainly by observational associations with cognition. In contrast, pharmacological-dose supplementation has been investigated as a potential intervention, but evidence for the prevention or treatment of DACD remains limited. Available intervention studies have primarily enrolled patients with T2DM and assessed cognitive performance without requiring standardized DACD diagnosis. The available interventional and observational studies are summarized in Table 2. A small, randomized, open-label, controlled trial of 74 adults with poorly controlled T2DM reported that vitamin E supplementation (600 mg/day for 3 months) improved total Mini-Mental State Examination (MMSE) scores and orientation, accompanied by reduced lipid peroxidation and increased antioxidant enzyme activity (63). These concurrent changes are consistent with the proposed antioxidant effects of vitamin E. However, the study design does not demonstrate that reduced oxidative stress caused cognitive improvement. In addition, the small sample size, short follow-up, lack of placebo blinding, and use of a single cognitive screening tool limit causal interpretation and generalizability. Conversely, another randomized, open-label trial found that vitamin E at 400 mg/day for 12 weeks did not significantly improve MMSE scores in patients with T2DM (64). This negative finding should also be interpreted with caution. Concomitant antidiabetic therapies should also be considered when interpreting these findings, as differences in background treatment may confound or modify the observed cognitive and metabolic responses to vitamin E. Safety assessment was limited in these short-duration trials. In particular, the 600 mg/day study did not report systematic monitoring of adverse events, liver function, bleeding complications, or medication interactions; therefore, the absence of reported serious adverse effects should not be interpreted as evidence of long-term safety. Because these intervention doses substantially exceeded usual dietary intake, they should be distinguished from dietary exposure and interpreted as high-dose or pharmacological-dose supplementation. For context, the European Food Safety Authority (EFSA) retains an adult tolerable upper intake level (UL) of 300 mg/day for α-tocopherol from all dietary sources, whereas the UL for supplemental α-tocopherol in the United States is 1,000 mg/day (65, 66). The exact vitamin E formulation was not clearly specified in these studies, which further limits interpretation of dose-safety relationships. Taken together, the available evidence remains insufficient to establish a clear clinical benefit of vitamin E in DACD. The gap between experimental and clinical findings may partly reflect differences in model complexity and study endpoints. Preclinical studies often use homogeneous models, short interventions, and biochemical outcomes, whereas DACD develops over years through interacting metabolic, vascular, and neurodegenerative processes. By clinical detection, some neuronal or microvascular injury may be only partly reversible; therefore, improvement in individual oxidative or inflammatory pathways may not translate into measurable cognitive benefit. Clinical heterogeneity may contribute to inconsistent findings. In T2DM, older age, female sex, and vascular comorbidities such as hypertension and cardiovascular disease have been associated with greater risk of cognitive impairment (67), while diabetic nephropathy and other renal disease may further increase cognitive vulnerability (68). Glycemic variability and recurrent hypoglycemia are also associated with higher dementia risk (69). Concomitant medications and polypharmacy may additionally influence cognitive outcomes and treatment safety in older adults (70).

**Table 2:** Clinical and observational evidence relevant to vitamin E and cognitive outcomes.

Study design	Population	Vitamin E formulation/Exposure	Dose	Duration	Cognitive outcome	Principal limitations	Safety findings	Indirect clinical evidence	Reference
Randomized, open-label, controlled trial	74 adults with poorly controlled T2DM	Vitamin E; formulation not specified	600 mg/day (Pharmacological-dose vitamin E supplementation)	3 months	Improved MMSE score and orientation	Small sample; short duration; no placebo blinding; MMSE only; no formal DACD diagnosis	Safety monitoring not systematically reported	Indirect clinical evidence	(63)
Randomized, open-label trial	Patients with T2DM	Vitamin E; formulation not specified	400 mg/day (Pharmacological-dose vitamin E supplementation)	3 months	No significant improvement in MMSE	Short duration; MMSE only; no formal DACD diagnosis; formulation unspecified	Limited safety assessment	Indirect clinical evidence	(64)
Cross-sectional observational study (NHANES 2011–2014)	U. S. adults aged ≥60 years	Dietary vitamin E intake	8.1 ± 5.2 mg/day	Cross-sectional	Higher intake associated with better cognition and lower odds of cognitive impairment	Cross-sectional design; recall bias; residual confounding; not diabetes-specific	Not applicable	Indirect observational evidence	(71)

Available interventional and observational studies provide indirect evidence relevant to DACD and are not specific to patients with formally diagnosed DACD.

Other human evidence is largely observational, focusing on associations between dietary vitamin E intake and cognitive outcomes in general older populations rather than DACD-specific cohorts. Data from the National Health and Nutrition Examination Survey (NHANES) 2011–2014 showed an L-shaped association between vitamin E intake and cognitive impairment in U. S. adults aged ≥ 60 years, with an inflection point of approximately 9.296 mg/day (71). However, the cross-sectional design, dietary-recall bias, and residual confounding preclude causal inference, and this value should not be considered a therapeutic target. Genetic background may also modify the association between vitamin E and cognitive outcomes. Among apolipoprotein E (APOE) ε4 carriers, higher food-derived α-tocopherol intake was associated with a slower rate of global cognitive decline, with participants in the highest quintile showing a 16.4% slower rate than those in the lowest quintile (72). Although this finding suggests that vitamin E may be more relevant in individuals with greater susceptibility to oxidative stress (73), it does not directly validate the proposed molecular mechanisms in DACD. Combined supplementation with vitamin E, omega-3 fatty acids, and carotenoids has been associated with improved cognitive performance (74), but the specific contribution of vitamin E cannot be determined. Consistent with this limitation, a recent systematic review concluded that the reported benefits were derived mainly from dietary-pattern and multi-nutrient studies, whereas clinical trials evaluating vitamin E alone or non-α-tocopherol isoforms remained scarce (10).

Dietary vitamin E is obtained from whole foods and is accompanied by other potentially beneficial nutrients. Nutritional supplementation provides vitamin E in a more concentrated and isolated form, whereas pharmacological doses substantially exceed usual dietary intake. Safety concerns are particularly relevant to pharmacological-dose supplementation. Safety data from small, short-duration T2DM trials are insufficient to establish long-term safety, particularly in older patients with multimorbidity and polypharmacy. A small study in obese patients with T2DM reported worsened insulin action and fibrinolytic parameters, representing a limited safety signal (75). High-dose α-tocopherol may impair coagulation and increase bleeding risk, especially with antiplatelet or anticoagulant therapy, and EFSA identified bleeding as the critical effect for setting the UL (65). Earlier meta-analysis suggested increased hemorrhagic-stroke risk (76), whereas more recent evidence found this mainly with combined antioxidant supplementation (77). Clinically significant hepatotoxicity is not an established adverse effect of vitamin E supplementation (78).

## 5 Challenges and future directions for clinical translation

Future studies should clearly distinguish prevention of cognitive decline in cognitively intact individuals with diabetes from treatment of established DACD and correction of documented vitamin E deficiency. Clinical translation requires adequately powered, multicenter, randomized, double-blind, placebo-controlled trials. Preclinical translation is also limited by chemically induced diabetes models, which incompletely reflect the metabolic, neurovascular, and age-related complexity of T2DM-associated DACD. Future studies should prioritize metabolically relevant T2DM models with direct cognitive assessment. To reduce clinical heterogeneity, studies should use standardized diagnostic criteria and stratify participants by cognitive stage, diabetes duration, glycemic control, baseline vitamin E status, genetic background, and concomitant medication use. Circulating vitamin E should be assessed together with total cholesterol and triglycerides, with both absolute and lipid-adjusted vitamin E concentrations reported to reduce misclassification of vitamin E status, particularly in patients with diabetic dyslipidemia.

Because tocopherol and tocotrienol isoforms differ in tissue retention, metabolism, bioavailability, and biological activity, trials should specify the isoform used and compare α-tocopherol with non-α-tocopherol isoforms and tocotrienol-containing preparations. Preclinical doses cannot be directly extrapolated to humans. Therefore, dose and treatment duration require systematic evaluation. Human dose-ranging studies with adequate follow-up are needed to identify clinically relevant and safe doses and to distinguish short-term biochemical responses from sustained cognitive benefits.

Future studies should analyze dietary intake, nutritional supplementation, and pharmacological-dose supplementation as distinct exposure categories. Future trials should use standardized neuropsychological batteries covering global cognition, executive function, processing speed, attention, and memory, together with functional outcomes, neuroimaging, and mechanistically relevant biomarkers. Before vitamin E supplementation can be recommended for DACD, studies should further establish long-term safety and clinically appropriate dose–response relationships, account for concomitant antidiabetic therapies as potential confounders or effect modifiers, and evaluate safety concerns related to antiplatelet or anticoagulant use.

## 6 Conclusion

The DACD involves interacting mechanisms, including oxidative stress, neuroinflammation, impaired synaptic plasticity, disrupted insulin signaling, and neurovascular dysfunction. Experimental studies suggest that specific vitamin E isoforms and preparations may modulate several of these pathways, providing a mechanistic rationale for further investigation (Figure 1). However, the evidence remains predominantly preclinical, and direct clinical evidence in formally diagnosed DACD is lacking. Small intervention studies in patients with T2DM have produced inconsistent cognitive outcomes, while observational associations between dietary vitamin E intake and cognition cannot establish therapeutic efficacy. Accordingly, current evidence does not support routine vitamin E supplementation for the prevention or treatment of DACD. Recommended intake should preferably be achieved through a balanced diet, whereas pharmacological-dose supplementation requires individualized assessment because of potential adverse effects and drug interactions. Future DACD-specific randomized controlled trials should clarify relevant vitamin E isoforms, doses, target populations, cognitive outcomes, and long-term safety. Until then, vitamin E should be regarded as a mechanistically plausible candidate rather than an established therapy.

**Figure 1:**
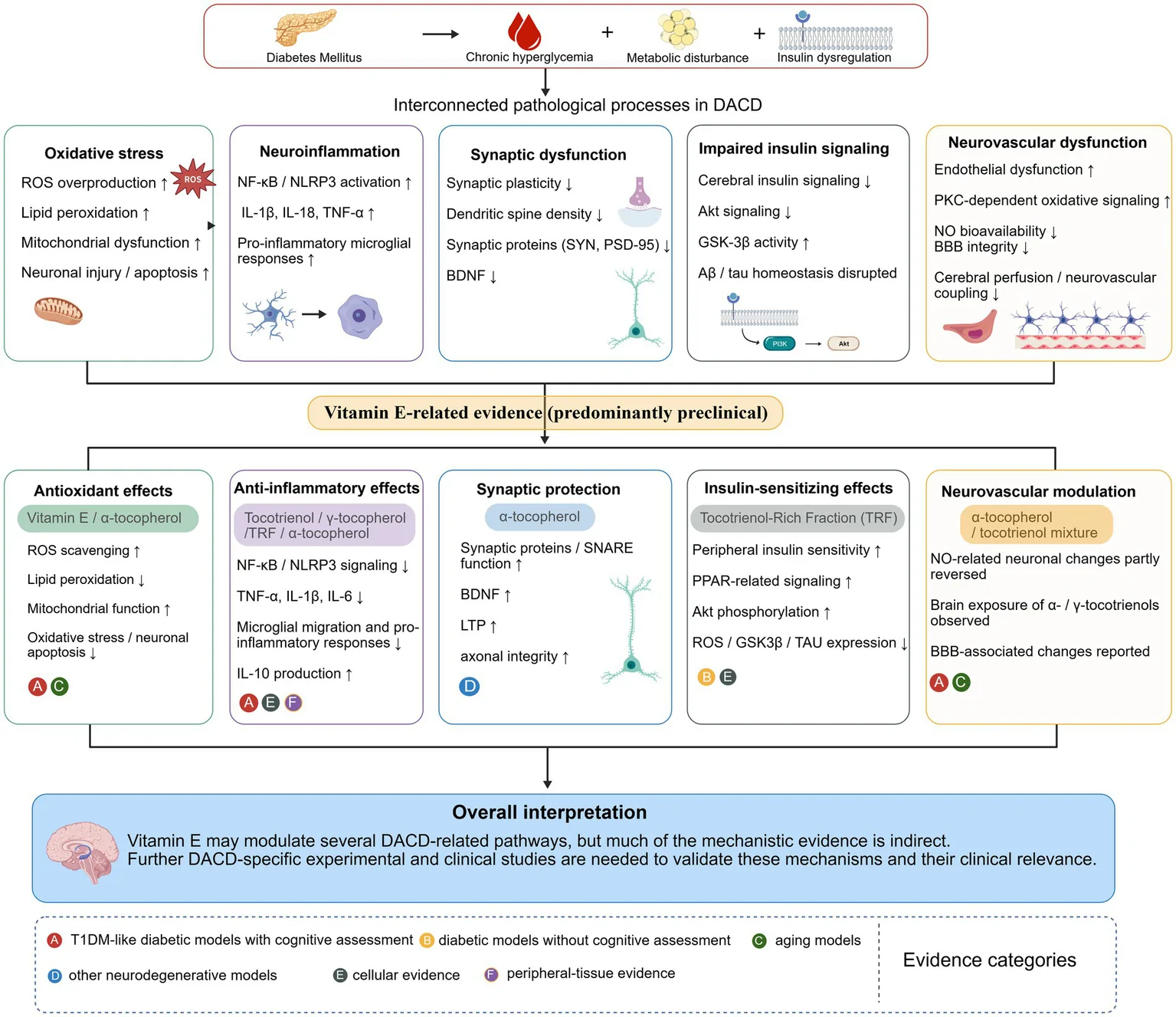
Proposed multi-target mechanisms of vitamin E in diabetes-associated cognitive dysfunction. The illustrated effects are derived from studies of different vitamin E isoforms and preparations and should not be interpreted as a uniform class effect across all forms of vitamin E. Created with BioGDP.com (79).
